# Partial Purification and Characterization of Exo-Polygalacturonase Produced by *Penicillium oxalicum* AUMC 4153

**DOI:** 10.3390/life12020284

**Published:** 2022-02-14

**Authors:** Shamsan A. Almowallad, Ghedeir M. Alshammari, Muneer M. Alsayadi, Naofel Aljafer, Ekram A. Al-Sanea, Mohammed Abdo Yahya, Laila Naif Al-Harbi

**Affiliations:** 1 Department of Food Science and Technology, Faculty of Agriculture and Food Science, Ibb University, Ibb P.O. Box 70270, Yemen; shamsanbio@gmail.com (S.A.A.); smuneer006@gmail.com (M.M.A.); 2 Department of Food Science and Nutrition, College of Food Science and Agriculture, King Saud University, Riyadh 11451, Saudi Arabia; aghedeir@ksu.edu.sa; 3 School of Biological and Marine Sciences, University of Plymouth, Plymouth PL4 8AA, UK; naofel.aljafer@plymouth.ac.uk; 4 Department of Biology, College of Sciences, Ibb University, Ibb P.O. Box 70270, Yemen; e.alsanea19@gmail.com

**Keywords:** Exo-polygalacturonase, *Penicillium oxalicum*, AUMC 4153, purification, characterization

## Abstract

Pectinase enzymes are important industrial enzymes having considerable applications in several industries, especially in food processing. Pectinases contribute 25% of global food enzyme sales. Therefore, the demand for a commercial enzyme with desirable characteristics and low production costs has become one of the great targets. Hence, this study aims to produce exo-polygalacturonase (exo-PG) using local fungal isolate *Penicillium oxalicum* AUMC 4153 by utilizing sugar beet manufacturing waste (sugar beet pulp) as a sole raw carbon source under shaken submerged fermentation, which is purified and characterized to optimize enzyme biochemical properties for industrial application. The purity of the obtained exo-PG was increased by about 28-fold, and the final enzyme yield was 57%. The partially purified enzyme was active at a broad range of temperatures (30–60 °C). The optimum temperature and pH for the purified exo-PG activity were 50 °C and pH 5. The enzyme was stable at a range of pH 3 to 6 and temperature 30–50 °C for 210 min. The values for *K*_*m*_ and *V*_*max*_ were 0.67 mg/mL, with polygalacturonic acid as substrate and 6.13 µmole galacturonic acid/min/mg protein, respectively. It can be concluded that purified exo-PG production by *P. oxalicum* grown on sugar beet waste is a promising effective method for useful applications.

## 1. Introduction

Increasing industrial pectinases applications and their market requires reducing commercial enzymes production costs and their activity loss. For this purpose, the selection of carbon sources and nitrogen sources with low cost and exploring new microbial isolates is a practical consideration. Besides that, the purification and characterization of the enzyme to obtain a commercial enzyme with desirable biochemical characteristics become valuable considerations for producers since they help optimize enzyme activity and recovery.

The enzymes that hydrolyze pectic substances are known as pectic enzymes, pectinases, or pectinolytic enzymes, which include pectinesterase (PE), polygalacturonase (PG), pectin lyase (PL), and pectate lyase (PAL) based on their mode of action. Polygalacturonases (PGases) are hydrolysis depolymerase with endo-PG (EC: 3.2.1.15) and exo-PG (EC: 3.2.1.67) activities [1,2,3,4]. The pectin hydrolases as polygalacturonases catalysis α-1,4-glycosidic linkage in pectic acid [5].

Microbial pectinases account for 25% of global food enzymes sales. Most commercial preparations of pectinases are produced from fungal sources [6,7]. The capability of synthesis pectinase enzymes is widespread among all microbial groups. However, fungi are preferred for industrial purposes because approximately 90% of their produced enzymes can be excreted into the culture medium [8]. Among fungi, *Aspergillus niger*, *Penicillium,* and *Rhizopus* have many advantages as enzyme producers. They are recognized as GRAS (generally regarded as safe) strains and yield extracellular products that can be easily extracted from their fermented medium [9]. The strains of *Aspergillus* and *Penicillium* are mainly used for exo-and endo-PGase production studies [10]. *Penicillium oxalicum* produces high polygalacturonase, pectinesterase, and pectin lyase [10]. Both submerged fermentation (SMF) and solid-state fermentation (SSF) techniques are widely used for PGase production by various types of microorganisms [11]. 

Pectinases contribute widely to several applications, especially in the food, textile and paper industries [12], and these are more suitable for coffee and tea fermentation, oil extraction, treatment of industrial wastewater, and degumming of plant fiber [13,14]. Polygalacturonase, pectinesterase, and pectin lyase have been used in fruit juice extraction, depectinization, and clarification, whereas pectinases combined with hemicellulase and cellulase are applied in cell wall decomposition and fruit juice extraction [15]. The largest industrial application of pectinases is in fruit juice extraction and clarification. Pectic substances contribute to juice viscosity and turbidity [2]. Recently, pectinase enzymes were used with cellulose enzyme in bioethanol production from superfine sugarcane bagasse [16].

Several previous studies have reported that numerous waste products from the food and agricultural industry contain pectin to reduce enzyme production costs, such as sugar beet pulp (SBP), citrus pulp pellets, orange peel, wheat bran, apple pomace, henequen pulp, lemon pulp, and other related sources, and have been used in inducing raw pectinase production by many microorganisms [17,18,19,20]. These enzymes not only provide an economically viable alternative; furthermore, they are environmental friends [21]. 

Sugar beet pulp (SBP), a by-product of the table sugar industry, is of low cost and is available in large amounts. In 2011, 271.6 million tons of sugar beets were produced globally [22]. It increased to 284.4 and 285.08 Mt in 2019 and 2021, respectively [23]. After sucrose extraction, it produces approximately 68 million tons of wet SBP or 17 million tons of dried biomass [24]. Sugar beet pulp is mainly composed of (% on dry basis) pectin, 28.7%; cellulose, 20%; hemicellulose, 17.5%; protein, 9.0%; and lignin 4.4% [25]. 

The purification of protein and the desired enzyme is crucial for designing and selecting pectinases. Moreover, the improved knowledge regarding biochemical properties of microbial pectinase is essential to have a better understanding of the pectinase mechanism of action, to obtain commercial enzymes, and to study the uses of the enzyme in various potential fields [26]. Pectinases from various sources of microorganisms have been purified to homogeneity by different series of methods such as salt and solvent precipitation, gel filtration chromatography, and ion-exchange chromatography [27,28]. The purification and characterization of the enzyme are important since optimal conditions of fermentation and enzyme recovery can reduce the production cost [29]. Besides that, the determination of enzyme kinetics could reveal the nature of the role of the catalytic mechanism in metabolism, enzyme activity, and inhibition control [30]. Therefore, we studied the production of exo-polygalacturonase by local fungal isolate *P. oxalicum* AUMC 4153 using dried sugar beet pulp as a sole raw carbon source under shaken submerged fermentation techniques. In addition, we investigated the purification of crude exo-PG using ammonium sulfate precipitation followed by cold acetone treatment and subsequent purification using gel filtration column chromatography (Sephadex G-200). This purified enzyme was characterized to optimize enzyme biochemical properties for industrial application, especially in the food and fruit juice industries (extraction and clarification).

## 2. Materials and Methods

### 2.1. Microorganisms

*P. oxalicum* AUMC 4153, highly pectinase-producing strains, were isolated from infected and decayed citrus fruits and identified at Assiut University Mycological Center (AUMC) as previously described by Almowallad [31]. 

### 2.2. Culturing Media

The stock cultures of fungal strains were maintained on potato dextrose agar medium slant in a refrigerator at 5 °C by periodic subculturing. Fungal spore suspensions used as inocula were prepared using fungal growth enhancement agar medium as described by Ismail [18]. Its composition was (g/L): glucose, 10; peptone, 5.0; yeast extract, 1.0; MgSO_4_, 0.5; KH_2_PO_4_, 1.0 and agar, 20. Slants were incubated at 30 °C for 4 days. Spores were harvested from the slants using sterile saline solution (0.9% *w*/*v*). The spore suspensions were adjusted to a final concentration of approximately 3 × 10^8^ spores per ml. The spore number was estimated by direct microscopic count using a hemocytometer.

### 2.3. Sugar Beet Pulp Powder

Sugar beet manufacturing waste is a good inducer for fungal pectinase production. The high pectin content (36.3 g/100 g on a dry weight basis) in this waste could be beneficial for pectinase enzymes production, as previously described by Almowallad et al.; Almowallad [32,33].

### 2.4. Chemicals

Dialysis tubing cellulose membrane (flat width 4.3 cm), (Sigma D9527, Germany). Folin ciocalteu’s phenol reagent (2 N) (Sigma-Aldrich F9252, USA) was used to estimate protein content. Bovine serum albumin (Sigma A2153, USA) was used to prepare the standard curve for calculating protein content. Sephadex G-200 (Pharmacia 17-0080-01, Sweden) was used for gel filtration chromatography. Dinitrosalicylic acid (Sigma D0550, USA) was used as a reagent to determine exo-PG activity. Polygalacturonic acid from orange (Sigma P3889, Spain) was used as a substrate to determine exo-PG activity. D-Galacturonic acid (Fluka 48280, Slovakia) was used to prepare the standard curve for calculating reducing sugar.

### 2.5. Cultivation of Fungi and Exo-PG Production by Submerged Fermentation

The fermentation broth medium used for fungal growth and enzyme production was prepared according to Paterson et al. [34], replacing pure pectin with sugar beet pulp powder (30 g/L). The medium composition was sugar beet pulp powder, 30; (NH_4_)_2_HPO_4_, 2; NH_4_H_2_PO_4_, 0.9; MgSO_4_, 0.1 and KCl, 0.5 g/L. Medium pH was adjusted to 7 (optimum pH for pectinase production by *P. oxalicum* AUMC 4153) [31]. Cultivation was carried out in a 250 mL Erlenmeyer flask; each flask contained 100 mL of sterile fermentation broth medium, which was inoculated with 1 mL of fungal spore suspension containing approximately 3 × 10^8^ spores per ml. Then, the flasks were incubated for 5 days at 30 °C under both static and shaken (150 rpm) submerged fermentation. All experiments were carried out in triplicate. At the end of the incubation period, each flask’s content was filtered through Whatman No.1 filter paper. The culture filtrates served as the crude enzyme source.

### 2.6. Assay for Exo-Polygalacturonase Activity (Exo-PG)

Exo-polygalacturonase activity in culture filtrate was determined colorimetrically by measuring the amount of liberated reducing sugars (as a result of enzyme action on the substrate) according to the method of dinitrosalicylic acid reagent (DNS) described by Miller [35]. One ml of 1% polygalcturonic acid (dissolved in 0.05 M sodium acetate buffer, pH 5.0) and 8.5 mL of sodium acetate buffer were added to 0.5 mL of culture filtrate. The reaction mixture was then incubated for 1 h at 45 °C in a controlled water bath [36]. Immediately after removing the samples from the water bath, 2 mL of 3.5-dinitrosalicylic acid reagent was added to 2 mL of the reaction mixture in a test tube. The test tube containing this mixture was heated in a boiling water bath for 15 min and then cooled under running tap water. One ml solution of 40% potassium sodium tartrate (Rochelle salt) was added to the mixture of reactants after the development of color and prior to cooling. The absorbance of the developed color of the mixture was measured at 575 nm using Shimadzu (UV-visible-1601PC) spectrophotometer. Similar reaction mixtures using heated inactive enzyme solutions (culture filtrate) were also prepared as a control. The exo-PG activity is expressed in units per milliliter of culture filtrate; one unit of exo-PG activity is defined as the amount of the enzyme that liberates one micromole (1 µmol) of galacturonic acid per minute under assay conditions. D-galacturonic acid was used as a standard [37].

### 2.7. Estimation of Protein Content

The protein content of the culture filtrates was determined by the method of Lowry et al. [38] using bovine serum albumin as standard.

### 2.8. Purification of Exo-Polygalacturonase 

Exo-polygalacturonase was purified from the culture filtrate of *P. oxalicum* AUMC 4153 grown on fermentation medium, which contained 3% (*w*/*v*) sugar beet pulp as a sole carbon source in shaken submerged fermentation at 150 rpm. The culture filtrates of fungal strain were centrifuged at 5000 rpm for 20 min at 4 °C to remove any mycelium and debris.

#### 2.8.1. Ammonium Sulfate Precipitation

Solid ammonium sulfate was slowly added to reach 20% saturation. Ammonium sulfate was added with gentle continuous stirring in an ice bath and was kept overnight at 4 °C. The precipitated protein was removed by centrifugation at 5000 rpm for 1 h at 4 °C ± 1. Ammonium sulfate was added to the supernatant to 80% saturation. The precipitated protein was removed by centrifugation at 5000 rpm for 1 h at 4 °C. The precipitated protein was dissolved in 25 mL of 0.1 M sodium acetate buffer pH 5 [39].

#### 2.8.2. Dialysis

The precipitate obtained after treatment with ammonium sulfate (80% saturation) was dialyzed in a cellulose membrane dialysis tube against 0.1 M sodium acetate buffer pH 5 overnight at 4 °C ± 1 under agitation, with a change of the buffer every four hours. The dialyzed enzyme solution was determined for exo-PG activity and protein content [40]. To check for the presence of sulfate, mix a small volume of the dialyzing fluid with an equal volume of a saturated barium chloride solution. If the mixture does not become cloudy, the dialysis can be considered complete [41]. Preparation of dialysis cellulose tubing membrane was carried out as described by Sigma-Aldrich chemical company, Germany.

#### 2.8.3. Acetone Precipitation

The dialyzed enzyme solution was cooled in an ice bath, and two equal volumes of cold acetone were added slowly, stirring gently so as to reach approximately 66% saturation. Then, it was left overnight at 4 °C ± 1. The precipitated protein was separated by centrifugation at 5000 rpm for 1 h at 4 °C. Then, it was dissolved in 9 mL (minimal volume) of 0.1 M sodium acetate buffer (pH 5). The activity of exo-PG and protein content of the fraction was determined at the end of this purification step [42].

#### 2.8.4. Gel Filtration Chromatography

The partially purified enzyme preparation was added to a Sephadex G-200 column (2.6 × 50 cm), previously equilibrated with 0.1 M sodium acetate buffer pH 5 and eluted with the same buffer at a flow rate of 0.3 mL per min. Fractions of 4 mL were collected, and both protein content and exo-PG activity were determined [39]. Protein content in the eluent was spectrophotometrically measured at 280 nm [43].

### 2.9. Characterization of Partially Purified Exo-Polygalacturonase

**Figure 1:**
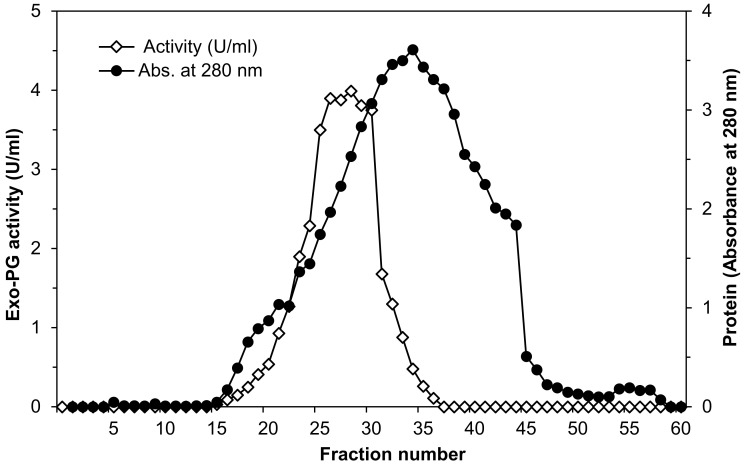
Elution diagram of *Penicillium oxalicum* AUMC 4153 exo-polygalacturonase on Sephadex G-200 column chromatography at a flow rate of 0.3 mL per min fraction volume (4 mL).

As in the previous step, only the fractions with maximal exo-PG specific activity (fractions 25–30, Figure 1) were pooled. This purified extract was used to determine the main characteristics of the enzyme.

#### 2.9.1. Effect of Temperature on the Exo-Polygalacturonase Activity

The enzyme activity was determined by incubating the reaction mixture (as described in the enzyme assay method) at different temperatures in the range from 25 to 70 °C. The optimum temperature for exo-PG activity was calculated by plotting enzymes activity against temperatures.

#### 2.9.2. Effect of pH on the Exo-Polygalacturonase Activity

The optimum pH for enzyme activity was determined by incubating the reaction mixture (as described in the enzyme assay method) at 50 °C and at different pH values 3, 4, 5, 6, 7, 8. These pH values were accessed using sodium acetate buffer (pH 3–5, 0.1 M), and potassium phosphate buffer (pH 6–8, 0.1 M). 

#### 2.9.3. Thermal Stability of Exo-Polygalacturonase Activity

This was tested by incubating the purified enzyme at various temperature degrees (30, 40, 50, and 65 °C) and for various periods (15, 30, 60, 90, 120, 150, 180, and 210 min). After cooling, the residual activities of enzymes were measured by the standard assay procedure at optimum temperature and pH (50 °C and pH 5) and calculated as relative activity.

#### 2.9.4. pH Stability of Exo-Polygalacturonase Activity

The effect of pH on the stability of purified exo-PG activity was studied. The enzyme was pre-incubated at different pH values (3, 4, 5, 6, 7 and 8) at 4 °C for various periods (15, 30, 60, 90, 120, 150, 180, and 210 min). These pH values were achieved by the application of different buffer systems: sodium acetate buffer (pH 3–5, 0.1 M) and potassium phosphate buffer (pH 6–8, 0.1 M). The residual activity of the enzyme was determined according to the standard assay method at optimum temperature and pH (50 °C and pH 5) and calculated as relative activity.

#### 2.9.5. Kinetics Constants of Exo-Polygalacturonase

The Michaelis–Menten constant (*K*_*m*_) and *V*_*max*_ of the partially purified exo-polygalacturonase from *P. oxalicum* AUMC 4153 were determined by measuring the velocities (µmole galacturonic acid/min/mg protein) at various concentrations of polygalacturonic acid (1–10 mg/mL), used as a substrate (S). The exo-PG activity was determined using the standard enzyme assay at optimum temperature and pH (50 °C and 5). The data were plotted according to the Lineweaver–Burk plot to calculate *K*_*m*_ and *V*_*max*_ values.

### 2.10. Statistical Analysis

All trials were accomplished in triplicate, and the means and standard deviations (SD) of the results were calculated. All data were statistically analyzed by analysis of variance (ANOVA) using general linear model GLM and Duncan’s multiple pot hoc tests by means of the SPSS V. 21 (SPSS Inc., Chicago, IL, USA). The differences were indicated as statistically significant at *p* ≤ 0.05 level. The Pearson correlation between variations was determined. 

## 3. Results

### 3.1. Purification of Exo-Polygalacturonase Enzyme

Exo-polygalacturonase was purified from the impurities and accompaniment compounds in the fermentation medium and the culture filtrate of *P. oxalicum* AUMC 4153 grown on sugar beet pulp by ammonium sulfate precipitation (20–80%), dialysis, cold acetone precipitation at 66% saturation, and finally by gel filtration chromatography using Sephadex G-200.

Table 1 summarizes the data on the purification of exo-PG at various purification steps. The specific activity of crude enzyme extract increased from 0.2 to 1.4 (seven-fold in purity) with 77% recovery of exo-PG activity by the ammonium sulfate precipitation step. The results presented in Table 1 also show that the cold acetone precipitation step resulted in 7.6-fold of purification with 63% recovery of exo-PG activity. While the other use of gel filtration chromatography purification step increased the enzyme purity by 3.7 times (28-fold purification) above obtained cold acetone precipitation, with 57% recovery of exo-PG activity.

Data of exo-PG purification using Sephadex G-200 column illustrated by the elution profile in Figure 1 shows that absorbance at 280 nm indicated that fractions 5–58 were found to have protein content and the maximum being found in fraction number 34. Exo-PG activity determinations showed that the fractions 15–36 were found to have exo-PG activity after, and the maximum was found in fraction number 28, being 3.99 U/mL. The fractions with maximal exo-PG activity were collected (fractions number 25–30). After purification, the specific activity increased from 0.2 to 5.63, equivalent to 28.2-fold compared to crude enzyme preparation with a 57% yield.

### 3.2. Characterization of Partially Purified Exo-Polygalacturonase

#### 3.2.1. Effect of Temperature on the Exo-Polygalacturonase Activity

**Figure 2:**
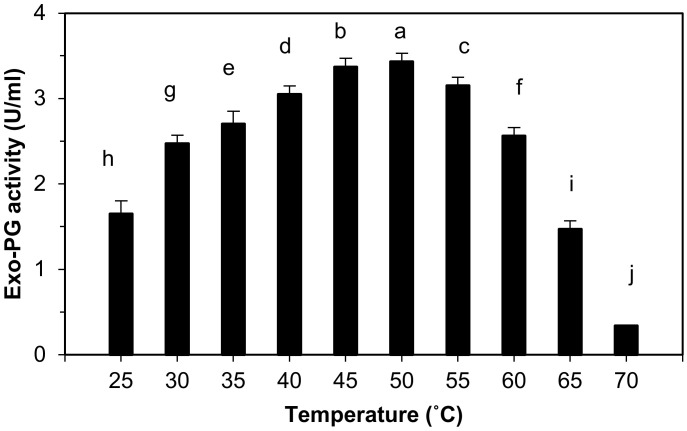
Effect of temperature on purified exo-polygalacturonase activity. The letters indicate that the mean difference is significant at the *p* ≤ 0.05 level.

Results represented graphically in Figure 2 indicate that the purified exo-PG activity was detected in the temperature range from 25 to 65 °C. Exo-PG enzyme activity increased significantly with increasing the incubation temperature of the enzyme reaction mixture up to 50 °C. The enzyme appeared as high activity at 30, 35, 40, 45, 50, 55, and 60 °C, but the highest activity (3.43 ± 0.01 U/mL) was obtained at 50 °C followed by 45 °C (3.37 ± 0.01 U/mL) and 55 °C (3.15 ± 0.01 U/mL). The finding demonstrated that the exo-PG activity increased gradually with the rising temperature until the optimum temperature (50 °C) was achieved. Meanwhile, the enzyme activity decreased sharply at above 65 °C with only 10% activity retained at 70 °C. 

The statistical analysis indicates that there are highly significant differences in the effects of temperature on the purified exo-PG activity between the groups and among the groups at *p* ≤ 0.05 level, and the multiple comparisons showed that the purified exo-PG activity was increased at 50 °C with high significant differences at *p* ≤ 0.05, followed by 45, 55, 40, 35, 60, 30, 25, 65 and 70 °C.

#### 3.2.2. Effect of pH on the Exo-Polygalacturonase Activity 

**Figure 3:**
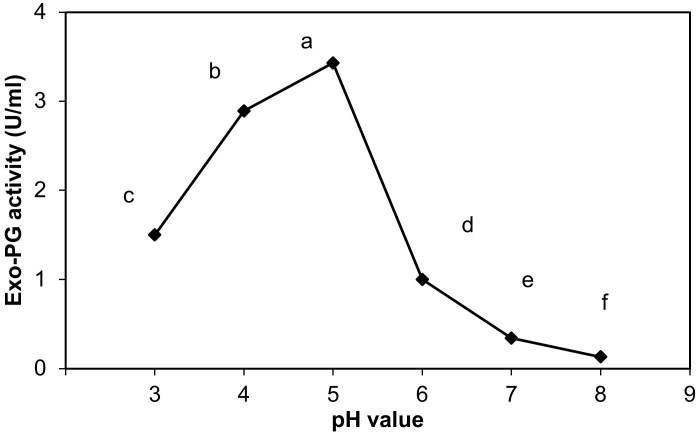
Effect of pH on purified exo-polygalacturonase activity. The letters indicate that the mean difference is significant at the *p* ≤ 0.05 level.

The effect of pH value (pH 3–8) on purified exo-PG activity was determined in the study, as shown in Figure 3, and demonstrates that the exo-PG activity strongly increased from 1.5 ± 0.06 to 3.43 ± 0.02 U/mL when the initial pH of the reaction mixture changed from 3 to 5. The most favorable pH value for exo-PG activity was found between 4 and 5 (2.89 ± 0.01 and 3.43 ± 0.02 U/mL), and the maximum activity differences were obtained at pH 5 and then 4 and 3, in order. Changing the pH value from 5 to 8 resulted in a sharp decrease in the exo-PG activity. These results proved that exo-PG is acidic.

Statistically, the pH changes appeared to be an effective factor on the purified exo-PG activity, with highly significant differences between the different pH. pH 5 significantly surpassed all other values in the exo-PG activity at level *p* ≤ 0.05, and pH 4 and 3 came in second and third, respectively, while the exo-PG activity decreased significantly at pH 6. Then, pH 7 and 8 recorded the most exo-PG activity declining at level *p* ≤ 0.05. 

#### 3.2.3. Thermal Stability of Exo-Polygalacturonase Activity

**Figure 4:**
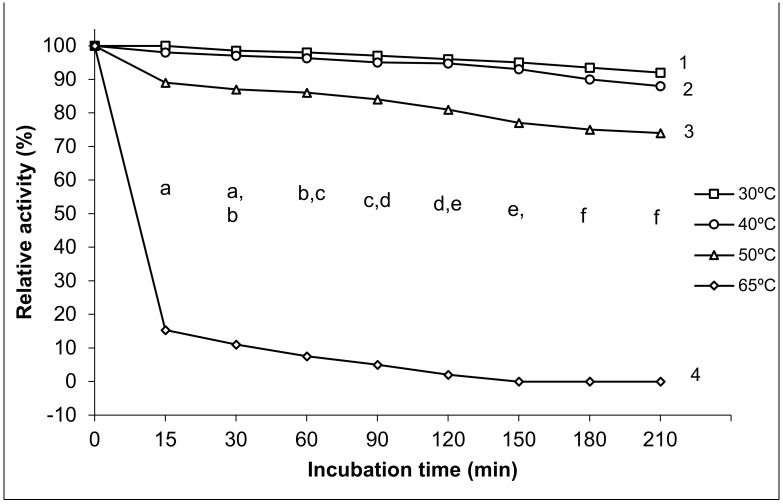
Thermal stability of purified exo-polygalacturonase activity. The different letters indicate that the mean difference is significant at the *p* ≤ 0.05 level among the incubation times. The different numbers indicate that the mean difference is significant at the *p* ≤ 0.05 level between the temperature degrees.

Four temperatures (30, 40, 50, and 65 °C) with the highest exo-PG activity were chosen to determine the purified exo-PG activity’s thermal stability. As observed in Figure 4, the partially purified exo-PG was highly stable at 30 and 40 °C and was relatively stable at 50 °C. It retained 96 ± 2.7% and 94.7 ± 3.5% of its original activity after 120 min. However, it retained about 92 ± 2.7% and 88 ± 3.5% of initial activity after 210 min at 30 and 40 °C, respectively. The enzyme activity slightly dropped for 50 °C with more than 81 ± 5.7% of activity retained after 120 min. However, it retained 74 ± 5.7% activity at the same temperature after 210 min of storage period. Incubation at 65 °C for 15 min resulted in activity loss by 75 ± 5.8% and became completely inactivated for more than 120 min at the same temperature. 

We found that the effects of incubation temperature degrees 30, 40, 50, 65 °C on the thermostability of purified exo-PG varied. The statistical analysis results showed significant differences in the thermal stability of purified exo-PG activity among the different temperature degrees at all incubation times and between the different temperature degrees at *p* ≤ 0.05 level. The changes in the incubation temperatures exhibited significant differences in the stability of the enzyme; little significant differences (*p* ≤ 0.05) were noted between 30 and 40 °C at all incubation periods, while the significant differences (*p* ≤ 0.05) were augmented with the increasing temperature, where 50 °C lowered the stability of the enzyme with significant differences (*p* ≤ 0.05) in comparison with that of 30 and 40 °C, and the most significant inhibition of the enzyme activity was found at the highest temperature of 65 °C. 

The multiple comparisons revealed that the thermal stability of purified exo-PG activity decreased significantly with the increase in time of incubation, the highest enzymatic relative activity was recorded at 15 min with high significant differences in comparison with the relative activity at all other incubation times, except for that of 30 min, which was similar with both periods of 15 and 60 min, while it surpassed all other incubation periods from 90 min and over with significant differences at *p* ≤ 0.05. Similarly, there were no significant differences shown between the time 120 and 150 min, and both of their prior and subsequent times, but significant differences appeared between the time 120 and both 180 and 210 min and between the time 150 and 210 min. However, no significant differences were recorded between the time 180 and 210 min at *p* ≤ 0.05. The thermal stability of the purified exo-PG was negatively correlated with both temperature and the incubation time; the Pearson correlation with temperature was 0.897 with high significance at the 0.01 level (two-tailed), and it was 0.106 with time, without significance.

#### 3.2.4. pH Stability of Exo-Polygalacturonase Activity

**Figure 5:**
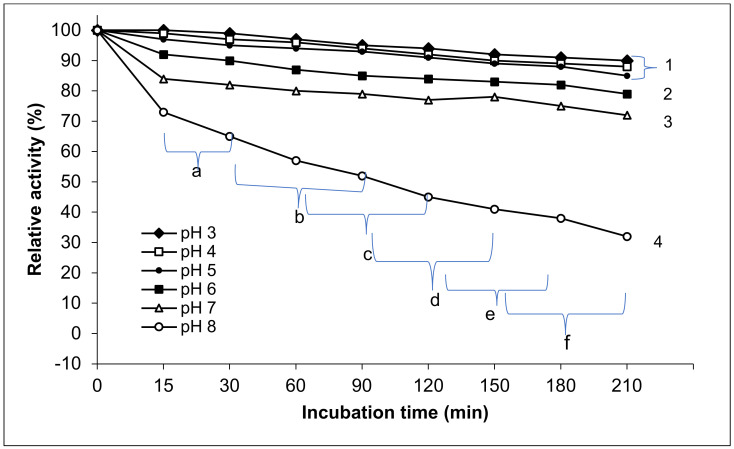
pH stability of purified exo-polygalacturonase activity. The different letters indicate that the mean difference is significant at the 0.05 level among the incubation times. The different numbers indicate that the mean difference between the pH values is significant at the *p* ≤ 0.05 level.

The purified exo-PG produced by *P. oxalicum* AUMC 4153 was stable at a range of pH 3–7, and its stability decreased above this range. Maximum stability was observed between pH 3–6. As shown in Figure 5, the enzyme retained 90 ± 3,7%, 88 ± 4.01%, 85 ± 4, 79 ± 4.3, and 72 ± 3.8% of its initial activity when incubated for 210 min at pH 3, 4, 5, 6, and 7, respectively. However, at pH 8, only 32% of its initial activity was retained.

The statistical results showed that the effect of different pH values on purified exo-PG stability appeared as significant differences between all tested pH values for various time intervals of storage (15–210 min) and within the pH values. The purified exo-PG activity exposed high stability at pH 3, 4, and 5 without significant differences at *p* ≤ 0.05 among them, while the significant differences appeared between these pH and all higher pH values. Consequently, the pH stability of purified exo-PG significantly (*p* ≤ 0.05) decreased with pH increasing from pH 6 to 7 and from 7 to 8, where the most significant decrease in enzyme stability was raised at pH 8. This paralleled with the extended incubation period. At the lowest 3 pH levels, the exo-PG stability was moderately significantly affected by time. However, the significant differences in the time effects on enzyme stability were augmented at pH 6, 7, and 8. Generally, the highest relative activity of the exo-PG was found at 15 and 30 min, with high significant differences in comparison with the other incubation periods at *p* ≤ 0.05, followed by 30, 60, and 90 min, but without significant differences between 60, 90 and 120 min, and there were likely no significant differences between each three subsequent times. The lowest significant differences in the relative activity of enzyme were at the periods 150, 180, and 210 min. The pH stability of the purified exo-PG negatively correlated with both pH and the incubation time. The Pearson correlation with pH was 0.804 with a high significance at the 0.01 level (two-tailed) and 0.313 with high significance at the 0.05 level (two-tailed).

#### 3.2.5. Kinetic Constants of Exo-Polygalacturonase Activity

**Figure 6:**
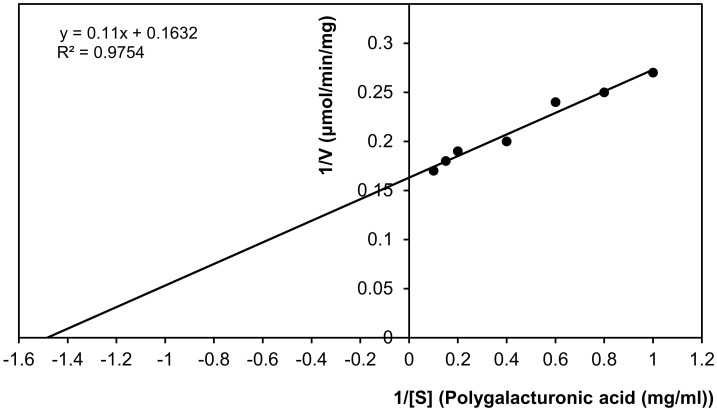
*K*_*m*_ and *V*_*max*_ values of purified exo-polygalacturonase. (Lineweaver–Burke plot).

According to the resulting Lineweaver–Burk plot, the values for *K*_*m*_ and *V*_*max*_ of the partially purified exo-polygalacturonase from *P. oxalicum* AUMC 4153 were 0.67 mg/mL, with polygalacturonic acid as substrate and 6.13 µmole galacturonic acid/min/mg protein, respectively. The results showed that the regression coefficient (R^2^) was 0.9754. Pearson correlation was 0.988 with a high significance at the 0.01 level (two-tailed), which indicated that the enzyme activity and the concentration of the substrate were positively correlated (Figure 6).

## 4. Discussion

### 4.1. Purification of Exo-Polygalacturonase Enzyme

Ammonium sulfate precipitation at level 80% was carried out in this study as an initial step, followed by acetone precipitation and gel filtration chromatography to purify crude extracellular polygalacturonase from *P. oxalicum* AUMC 4153. A high concentration of ammonium sulfate was used since the salting-out process depends on the protein’s hydrophobicity. Thus, a high salt concentration promotes the aggregation of hydrophobic patches on the protein surface [44]. Furthermore, the addition of salt in high concentration reduces the electrostatic repulsion between similar-charged groups at the protein surface. It disturbs the structure of water molecules around the protein, making the aqueous salt solution a poor solvent for proteins, which precipitate out [45].

The results of this study are somewhat similar to those obtained from many researchers implementing the ammonium sulfate precipitation method as the first step in their purification process. For instance, Jalil and Ibrahim [46] reported a 14-fold of purification and 86% yield of exo-polygalacturonase from *Aspergillus niger*, and LFP-1 were attained at 80% ammonium sulfate saturation. Coelho et al. [47] found a three-fold purification and 68.3% yield of exo-polygalacturonase from *Aspergillus niger* attained at 70% ammonium sulfate saturation. In addition, Mansour [42] obtained 3.4-fold of purification levels with 83% recovery of enzyme activity when he used ammonium sulfate at 78% saturation to precipitate exo-polygalacturonase from *Aspergillus niger*. Meanwhile, a 4.6-fold of purification and 89.6% enzyme yield were attained at 70% ammonium sulfate saturation, as reported by Doukani [48].

In agreement with this study’s results regarding enzyme purification by acetone (66%), a 5.3-fold of purification levels with 68% recovery of enzyme activity was obtained when the authors of [49] used 30–60% cold acetone to precipitate exo-polygalacturonase from *Botrytis cinerea* T91-1. While Mansour [42] obtained 3.5-fold of purification levels with 60% recovery of enzyme activity when using cold acetone at 66% saturation to precipitate exo-polygalacturonase from *Aspergillus niger* No. 36 after the ammonium sulfate precipitation step. Conversely, Kaur et al. [50] purified exo-polygalacturonase from thermophilic mold *Sporotrichum thermophile* by using acetone precipitation with 22.18% recovery of the enzyme at 19-fold of purification. In addition, Khatri et al. [51] reported that the specific activity of the crude enzyme obtained after precipitation with 66% cold acetone increased by 1.2-fold with a 19% yield when they purified exo-polygalacturonase partially from *Aspergillus niger* MCAS2.

The purification of exo-PG by gel filtration chromatography on Sephadex G-200 after ammonium sulfate and acetone precipitation steps increased the enzyme-specific activity equivalent to 28-fold compared to crude enzyme preparation with 57% yield. These results are somewhat similar to those reported by Doukani [48], who purified exo-polygalacturonase from *Aspergillus niger* U-86 using ammonium sulfate fractionation followed by gel filtration Sephadex G-75 and obtained a purification of 6.1-fold with an enzyme yield of 64%. In addition, Mansour [42] purified exo-polygalacturonase from *Aspergillus niger* No. 36 by ammonium sulfate fractionation (78% saturation), acetone precipitation, and gel filtration on Sephadex G-100 with a purification fold of 11.9 and enzyme yield of 40%. Meanwhile, Khatri et al. [51] found that the specific activity increased by 8.5-fold with 16% yield by gel filtration on Sephadex G-75 after precipitation with 66% cold acetone when they purified exo-polygalacturonase partially from *Aspergillus niger* MCAS2. It was found that the polygalacturonase from *Aspergillus niger* grown on banana peels was purified up to 42-fold [52]. However, these variations in purification fold and specific enzyme activity purified by gel filtration chromatography may depend on the microbial strains and substrate [46]. Gel filtration chromatography referred to as size exclusion chromatography is a chromatography technique that allows for the separation of macromolecules based on their hydrodynamic size, whereby the smaller molecules can access the higher number of pores and stay inside the column longer, while the larger molecules elute earlier because they can only penetrate larger pores [53]. 

### 4.2. Characterization of Partially Purified Exo-Polygalacturonase

#### 4.2.1. Effect of Temperature on Exo-Polygalacturonase Activity

The data of this study are in accordance with those reported by Lee et al. [49], Barense et al. [54], Chellegatii et al. [55], and Ramachandran [39], in which they demonstrated that the optimum temperature for the activity of exo-polygalacturonase produced from *Penicillium frequentans* and *Penicilium citrinum* was 50 °C. In addition, Jalil and Ibrahim [46], Khatri et al. [51], and Ahmed et al. [56] found that exo-polygalacturonase produced by *Aspergillus niger* LFP-1, *Aspergillus niger* MCAS2, and *Aspergillus niger*, respectively, was optimally active at 50 °C 

Conversely, several previous studies have indicated that the optimum temperature for the exo-polygalacturonase activity could differ, as shown by Kaur et al. [50], who found that exo-polygalacturonase produced from thermophilic mold *Sporotrichum thermophile* was optimally active at 55 °C. It was 40 °C for the same enzyme produced from *Aspergillus niger* MIUG 16 [57]. The decrease in enzyme activity at the high temperature degree attributed to the enzymes’ denaturation.

#### 4.2.2. Effect of pH on the Exo-Polygalacturonase Activity

The effect of different pH values on purified exo-PG activity in this study proved that exo-PG is acidic since pectinase enzymes are classified into alkaline and acidic pectinases depending on pH demand for optimum enzymatic activity [58]. The pH value greatly affected the enzyme activity, as the binding of substrate and catalyst is affected by the distribution of charge on enzyme molecules and substrate [59].

Thus, the present study results are in line with those revealed by Lee et al. [49], who recorded that optimum pH for exo-polygalacturonase activity from *Botrytis cinerea* T91-1 was 4 and 5. Chellegatii et al. [55] found that the optimum pH for the activity of exo-polygalacturonase from *Penicillium frequentans* was 3.9. In addition, Ramachandran [39] and Gewali et al. [60] noted that the optimum pH for the activity of exo-polygalacturonase produced from *Penicilium citrinum* was 4.5. A similar observation was reported by Ahmed and Sohail [61], who found that the pectinase has highly active at acidic pH in the range of 3 to 5.5. Conversely, these results differed from Kaur et al. [50] and Khatri et al. [51]. They reported that exo-polygalacturonase from thermophilic mold *Sporotrichum thermophile* and *Aspergillus niger* MCAS2 was optimally active at pH 7.0 and pH 8.2, respectively.

The inhibition of enzyme activity by the increasing of pH can be attributed to the changes in the hydrogen/hydroxyl ions concentration. In addition, they inhibit the enzyme by the denaturation, precipitation, blocking of the effect sites and binding of some side-chain functional groups of the enzyme, which may affect the ionic equilibrium, liberation of some inhibitors, and the binding of enzyme activators, in addition to the potential effects on the substrates and water activity of the media. 

#### 4.2.3. Thermal Stability of Exo-Polygalacturonase Activity

The results of the present study, confirmed by Jalil and Ibrahim [46], concluded that the exo-polygalacturonase from A. niger LFP-1 grown on pomelo peels was more stable at a temperatures range of 40 to 50 °C after 90 min of storage period. These obtained results also agree with those observed by Maciel et al. [62]. They studied the thermal stability of purified pectinase from *Aspergillus niger* URM4645 grown on forage palm and found that the enzyme activity was stable at 50 °C and deactivated at higher temperatures. The exo-polygalacturonase from *Aspergillus niger* No. 36 was completely destroyed by heating at 70 °C for 20 min [42]. Moreover, in agreement with that noted by Kumar and Palanivelu [63], the exo-polygalacturonase from thermophilic fungus *Thermomyces lanuginosus* was completely stable at room temperature (32 ± 3 °C) and retained about 50% activity at 50 °C for 6 h. 

The thermal inactivation of enzymes is nearly always due to enzyme denaturation. Pectinases from Aspergillus strains have been described to be susceptible to denaturation at a temperature above 50 °C [64]. Following the present study results, enzyme activity becomes unstable at higher temperature, which might result in hydrolysis of peptide bonds, destruction of disulfide bonds, oxidation, and delamination of the amino acid side chains of protein molecules [52].

#### 4.2.4. pH Stability of Exo-Polygalacturonase Activity

The findings of this study are in close agreement with those obtained by Doukani [48], who found that the purified exo-polygalacturonase from *Aspergillus niger* U-86 was stable at acidic pH (3–6), and its stability decreased above this range. In addition, Lee et al. [49] recorded that exo-polygalacturonase activity from *Botrytis cinerea* T91-1 was stable up to 12 h in the range of pH 3 to 8. Maximum stability of exo-polygalacturonase from *Penicilium citrinum* was observed between pH 4.0–5.5 [39].

#### 4.2.5. Kinetic Constants of Exo-Polygalacturonase Activity

The present study demonstrated that the partially purified exo-PG from *P. oxalicum* AUMC 4153 has a high affinity toward the substrate due to its lowest *K*_*m*_ value (0.67 mg/mL). Furthermore, the enzyme has the highest utility of polygalacturonic acid (substrate), as a result of its highest *V*_*max*_. The finding showed that a small quantity of the enzyme would digest a considerably high substrate.

Kinetics constants obtained in this study are rather closed to those observed by Mansour [42], who found that the *K*_*m*_ and *V*_*max*_ values of exo-polygalacturonase activity from *Aspergillus niger* No. 36 were 1.136 mg/mL, with sodium polypectate as the substrate and 5.26 µmol/min/mg. The *K*_*m*_ and *V*_*max*_ values of exo-polygalacturonase from *Penicilium citrinum* were 0.1 mg/mL, with polygalacturonic acid as substrate and 5 µmol/min/mg, respectively [39]. At the same time, Dinu et al. [57] found the *K*_*m*_ value of purified exo-polygalacturonase from *Aspergillus niger* MIUG 16, with sodium polygalacturonate as the substrate at 0.94 mg/mL. Conversely, the present results differ from those described by Lee et al. [49], who recorded that *K*_*m*_ and *V*_*max*_ values of exo-polygalacturonase from *Botrytis cinerea* T91-1 were 0.33 mg/mL, with polygalacturonic acid and 28.6 nmol/min/mg, respectively. Kaur et al. [50] found that *K*_*m*_ and *V*_*max*_ values (for pectin) of exo-polygalacturonase from thermophilic mold *Sporotrichum thermophile* were 0.416 mg/mL and 0.52 µmol/min/mg, respectively. These differences in *K*_*m*_ and *V*_*max*_ values of exo-polygalacturonase may result in fungal isolates, fermentation technique (solid state or submerged fermentation) and purification steps (partial or high purification), which are used in production and purification of the enzymes. In addition, these differences in *V*_*max*_ value of exo-polygalacturonase may result in the determination methods of enzyme-specific activity in units (µmol/mg or nmol/mg or µgram/mg).

## 5. Conclusions

Exo-polygalacturonase from *P. oxalicum* AUMC 4153 grown on sugar beet pulp was purified successfully with a maximum activity of almost 28 higher than crude enzyme and high enzyme recovery. This purified exo-PG obtained with desirable biochemical properties can be used in various industrial biotechnological applications, especially in the food and juice industries. It is not enough to produce high-activity purified enzymes. However, the reduction in production cost and increase in enzyme yield are also required to meet the growing demand for pectinase enzymes in several industrial applications. Thus, additional studies are recommended to optimize pectinase enzyme production and purification from new microbial isolates using other industrial waste.

## Figures and Tables

**Table 1:** Summary of purification steps of exo-polygalacturonase from *P. oxalicum* AUMC 4153.

Purification Steps	Total Activity (Units)	Total Protein (mg)	Specific Activity (U/mg)	Yield (%)	Purification (Fold)
Crude enzyme	141	699	0.2	100	1
Ammonium sulphate precipitation 20–80%	109	78	1.4	77	7
Acetone precipitation 66%	89	58.5	1.52	63	7.6
Gel filtration chromatography	81	14.4	5.63	57	28

## Data Availability

The datasets used and analyzed during the current study are available from the corresponding author on reasonable request.

## References

[1] Alkorta I., Garbisu C., Liama M.J., Serra J.S. (1998). Industrial applications of pectic enzymes: A review. Process. Biochem..

[2] Kashyab D.R., Vohra P.K., Chopra S., Tewari R. (2001). Application of pectinases in the commercial sector: A review. Bioresour. Technol..

[3] Kuhad R.C., Kapoor M., Rustagi R. (2004). Enhanced production of an alkaline pectinase from *Streptomyces* sp. RCK-SC by whole-cell immobilization and solid-state cultivation. World J. Microbiol. Biotechnol..

[4] Li Z.M., Jin B., Zhang H.X. (2008). purification and characterization of three alkaline endo- polygalacturonase from a newly isolated *Bacillus gibsonii*. Chin. J. Process. Eng..

[5] Palagiri S., Mayukha M., Sagar G., Chourasiya R., Sibi G. (2019). Production of Pectinases and Pectinolytic Enzymes: Microorganisms, Cultural Conditions and Substrates. Adv. Biotechnol. Microbiol..

[6] Singh S.A., Ramakrishna M., Rao A.G.A. (1999). Optimization of down-stream processing parameters for the recovery of pectinase from the fermented broth of *Aspergillus carbonarious*. Process. Biochem..

[7] Prathyusha K., Suneetha V. (2011). Bacterial pectinases and their potent biotechnological application in fruit processing/juice production industry: A review. J. Phytol. Res..

[8] Solis S., Flores M.E., Huitron C. (1990). Isolation of endo- polygalacturonases hyperproducing mutant of *Aspergillus* sp. CH-Y-1043. Biotechnol. Lett..

[9] Blanco P., Sieiro C., Villa T.G. (1999). Production of pectic enzymes in yeast. FEMS Microbiol. Lett..

[10] Favela-Torres E., Volke-Sepulveda T., Viniegra-Gonzalez G. (2006). Production of hydrolytic depolymerising pectinases: A review. Food Technol. Biotechnol..

[11] Jing L., Wang B., Long F., Yue B., Zhang M., Zng Q. (2008). Study on the composition of substrate and ferment condition of *Penicillium oxalicum* Curri & Thom production pectinase. J. Shenyang Agric. Univ..

[12] Reid I., Ricard M. (2000). Pectinase in paper making Solving retention problems in mechanical pulps bleached with hydrogen peroxide. Enzym. Microb. Technol..

[13] Kapoor M., Beg Q.K., Bushan B., Sing K., Dadhich K.S., Hoondal G.S. (2001). Application of an alkaline and thermostable polygalacturonase from *Bacillus* sp. MG-CP-2 in degumming of ramie (*Boehmeria nivea*) and sunn hemp (*Crotaloria juneea*) bast fibers. Process. Biochem..

[14] Hoondal G.S., Tiwari R.P., Tewari R., Dahiya N., Beg O.K. (2002). Microbial alkaline pectinases and their industrial applications: A review. Appl. Microbiol. Biotechnol..

[15] Voragen A., Wolters H., Verdonschot-Kroef T., Rombouts F.M., Pilnik W. (1986). Effect of Juice-Releasing Enzymes on Juice Quality. Proceedings of the International Fruit Juice Symposium.

[16] Li J., Zhou P., Liu H., Lin J., Gong Y., Xiao W., Liu Z. (2014). Monosaccharides and ethanol production from superfine ground sugarcane bagasse using enzyme cocktail. Bioresources.

[17] Said S., Fonseca M.G.V., Siessere V. (1991). Pectinase production by *Penicillium frequentans*. World J. Microbiol. Biotechnol..

[18] Ismail A.S. (1996). Utilization of orange peels for the production of multienzyme complexes by some fungal strains. Process. Biochem..

[19] El-Sheekh M.M., Ismail A.S., El-Abd M.A., Hegazy E.M., El-Diwany A.I. (2009). Effective technological pectinases by *Aspergillus carneus* NRC1 utilizing Egyptian orange juice industry scarps. Int. Biodeterior. Biodegrad..

[20] Heerd D., Diercks S., Fernandes-Lahore M. (2014). Efficient polygalacturonase production from solid-state culture of *Aspergillus sojae* under optimized conditions. Springer Plus.

[21] Viikari L., Tenkanen M., Suuranakki M. (2001). Biotechnology Set.

[22] (2011). FAO. http://www.fao.org.

[23] OECD/FAO, 2021. OECD-FAO Agricultural Outlook (Edition 2021). https://www.oecd-ilibrary.org/content/data/4bde2d83-en.

[24] Foster B.L., Dale B.E., Doran-Peterson J.B. (2001). Enzymatic hydrolysis of ammonia-treated sugar beet pulp. Appl. Biochem. Biotechnol..

[25] Jacob N. (2009). Pectinolytic enzymes. Biotechnology for Agro-Industrial Residues Utilization.

[26] Zhang C.H., Li Z.M., Peng X.W., Jia Y., Zhang H.X., Bai Z.H. (2009). Separation, purification and characterization of three endo- polygalacturonase from newly isolated *Penicillum oxalicum*. Chin. J. Process. Eng..

[27] Lekha P.K., Lonsane B.K. (1991). Comparative titres, location and properties of tannin acyl hydrolase produced by *Aspergillus niger* PKL104 in solid-state, liquid surface and submerged fermentations. Process. Biochem..

[28] Gummadi S.N., Panda T. (2003). Purification and biochemical properties of microbial pectinases: A review. Process. Biochem..

[29] Siddiqui M.A., Pande V., Arif M. (2012). Production, Purification, and Characterization of Polygalacturonase from *Rhizomucor pusillus* Isolated from Decomposting Orange Peels. Enzym. Res..

[30] Meena K.K., Jaipal M.K., Singh U. (2015). Production kinetics and characterization of pectinase enzyme from *Aspergillus niger*. South Asian J. Food Technol. Environ..

[31] Almowallad S.A. (2008). Studies on Fungal Pectinase Enzymes. Master’s Thesis.

[32] Almowallad S.A., Aljobair M.O., Alkuraieef A.N., Aljahani A.H., Alsuhaibani A.M., Alsayadi M.M. (2022). Utilization of agro- industrial orange peel and sugar beet pulp waste for fungal endo-polygalacturonase production. Saudi J. Biol. Sci..

[33] Almowallad S.A. (2012). Studies on Partial Purification and Characterization of Fungal Pectinases Enzymes and Their Application in Food Processing. Ph.D. Thesis.

[34] Paterson R.R., Bridge P.D., Crosswaite M.J., Howksorth D.L. (1989). A reapprasial of terverticillate pencillia using biochemical, physiological and morphological features III. An evaluation of pectinase and amylase isenozymes for species characterization. J. Gen Microbiol..

[35] Miller G.L. (1959). Use of dinitrosalicylic acid reagent for the determination of reducing sugar. Anal. Chem..

[36] Baracat M.C., Valentim C., Muchovej J.J., Silvia D.O. (1989). Selection of pectinolytic fungi for degumming natural fibers. Biotechnol. Lett..

[37] Moyo S., Gashe B.A., Collison E.K., Mpuchane S. (2003). Optimizing growth conditions for the pectinolytic activity of *Kluyveromyces wickerhamii* by using response surface methodology. Int. J. Food Microbiol..

[38] Lowry O.H., Rosebrough N.J., Farr A.L., Randall R.J. (1951). Protein measurement with the Folin phenol reagent. J. Biol. Chem..

[39] Ramachandran S. (2005). Isolation, Purification and Characterization of Pectinase from *Penicillium Citrinum* of Pectinase: Characteristics and Applications. Ph.D. Thesis.

[40] Hara T., Lim J.Y., Fujio Y., Ueda S. (1984). Purification and some properties of exo- polygalacturonase from *Aspergillus niger* cultured in the medium containing Satsuma mandarin peel. Nipp. Shok. Kog. Gakk..

[41] Hebert G.A., Pelham P.L., Pittman B. (1973). Determination of the optimal ammonium sulfate concentration for the fractionation of Rabbit, Sheep, Horse and Goat Antisera. Appl. Microbiol..

[42] Mansour S.M. (1996). Studies on Pectinolytic Enzymes of Fungi. Ph.D. Thesis.

[43] Arbaisah S.M., Asbi B.A., Junainah A.H., Jamilah B. (1997). Purification and properties of pectinesterase from soursop (*Anona muricata*) pulp. Food Chem..

[44] Farinas C.S., Scarpelini L.M., Miranda E.A., Bertucci Neto V. (2011). Evaluation of operational parameters on the precipitation of endoglucanase and xylanase produced by solid state fermentation of *Aspergillus niger*. Braz. J. Chem. Eng..

[45] Kumar A., Galaev I.Y., Mattiasson B., Hutti-Kaul R., Mattiasson B. (2003). Precipitation of proteins: Nonspecific and specific. Isolation and Purification of Proteins.

[46] Jalil M.T.M., Ibrahim D. (2021). Partial purification and characterization of pectinase produced by *Aspergillus niger* LFP-1 grown on pomelo peels as a substrate. Top. Life Sci. Res..

[47] Coelho M.A.Z., Medronho R.C., Leite S.G.F., Couri S. (1995). Partial purification of a polygalacturonase produced by solid state cultures of *Aspergillus niger* 3T5P. Braz. J. Microbiol..

[48] Doukani K. (2009). Microbial Production of Pectinase: Characteristics and Applications. Ph.D. Thesis.

[49] Lee T.H., Kim B.Y., Chung Y.R., Lee S.Y., Lee C.W., Kim J.W. (1997). Purification and characterization of an exo- polygalacturonase from *Botrytis cinerea*. J. Microbiol..

[50] Kaur G., Kumar S., Satyanarayana T. (2004). Production, characterization and application of a thermostable exo- polygalacturonase of thermophilic mould *Sporotrichum thermophile* Apinis. Bioresour. Technol..

[51] Khatri B.P., Bhattarai T., Shrestha. S., Maharjan J. (2015). lkaline thermostable pectinase enzyme from *Aspergillus niger* strain MCAS2 isolated from Manaslu Conservation Area, Gorkha, Nepal. Springer Plus.

[52] Ire F.S., Vinking E.G. (2016). Production, purification and characterization of polygalacturonase from *Aspergillus niger* in solid state and submerged fermentation using banana peels. J. Adv. Biol. Biotechnol..

[53] Raak N., Abbate R.A., Lederer A., Rohm H., Jaros D. (2018). Size separation techniques for the characterization of cross-linked casein: A review of methods and their applications. Separations.

[54] Barense R.I., Chellegatti M.A.S., Fonseca M.J.V., Said S. (2001). Partial Purification and characterization of exo- polygalacturonase II and III from *Penicillium frequentans*. Braz. J. Microbiol..

[55] Chellegatti M.A.S., Fonseca M.J.V., Said S. (2002). Purification and partial characterization of exo- polygalacturonase from *Penicillium frequentans*. Microbiol. Res..

[56] Ahmed I., Zia M.A., Hussain M.A., Akram Z., Naveed M.T., Nowrouzi A. (2016). Bioprocessing of citrus waster peel for induced pectinase production by *Aspergillus niger*; its purification and characterization. J. Radiat. Res. Appl. Sci..

[57] Dinu D., Nechifor M.T., Stoian G., Costache M., Dinischiotu A. (2007). Enzyme with new biochemical properties in the pectinolytic complex produced by *Aspergillus niger* MIUG 16. J. Biotechnol..

[58] Sharma D.C., Satyanarayan T., Reddy M.S., Khanna S. (2004). Production and application of pectinolytic enzymes of *Sporotrichum thermopohile* and Bacillus pumilus. Biotechnolgical Approaches for Sustainable Development.

[59] Kulkarni N.S., Jaiswal J.V., Bodhankar M.G. (2007). Influence of agro-waste amendment on soil microbial population in relation to plant growth response. J. Environ. Biol..

[60] Gewali M., Maharjan J., Thapa S., Shrestha J.K. (2007). Studies on polygalacturonase from *Aspergillus flavus*. Sci. World.

[61] Ahmed A., Sohail M. (2020). Characterization of pectinase from *Geotrichum candidum* AA15 and its potential application in orange juice clarification. J. King Saud Univ. Sci..

[62] Maciel M.D.C., Herculano P.N., Porto T.S., Teixeira M.F.S., Moreira K.A., Souza-Motta C.M. (2011). Production and partial characterization of pectinases from forage palm by *Aspergillus niger* URM4645. Afr. J. Biotechnol..

[63] Kumar S.S., Palanivelu P. (1999). Purification and characterization of an exo- polygalacturonase from the thermophilic fungus, *Thermomyces lanuginosus*. World J. Microbiol. Biotechnol..

[64] Galiotou-Panayotou M.P.R., Kapantai M., Kalantzi O. (1997). Growth conditions of *Aspergillus* sp. ATHUM-3428 for polygalacturonase production. Appl. Microbiol. Biotechnol..

